# Primary tumor location as a predictor of the benefit of palliative resection for colorectal cancer with unresectable metastasis

**DOI:** 10.1186/s12957-017-1198-0

**Published:** 2017-07-27

**Authors:** Rong-xin Zhang, Wen-juan Ma, Yu-ting Gu, Tian-qi Zhang, Zhi-mei Huang, Zhen-hai Lu, Yang-kui Gu

**Affiliations:** 1 Department of Colorectal Surgery, Sun Yat-sen University Cancer Center, Guangzhou, 510060 Guangdong People’s Republic of China; 2 State Key Laboratory of Oncology in South China, Guangzhou, 510060 Guangdong People’s Republic of China; 3 Collaborative Innovation Center of Cancer Medicine, Guangzhou, China; 4 Medical Record Department of The First Affiliated Hospital of Sun Yat-sen University, Guangzhou, 510060 Guangdong People’s Republic of China; 5 Microinvasive Interventional Department, Cancer Center, Sun Yat-sen University, Guangzhou, 510060 Guangdong People’s Republic of China

**Keywords:** Colorectal cancer, Unresectable liver metastases, Primary tumor site

## Abstract

**Background:**

It is still under debate that whether stage IV colorectal cancer patients with unresectable metastasis can benefit from primary tumor resection, especially for asymptomatic colorectal cancer patients. Retrospective studies have shown controversial results concerning the benefit from surgery. This retrospective study aims to evaluate whether the site of primary tumor is a predictor of palliative resection in asymptomatic stage IV colorectal cancer patients.

**Methods:**

One hundred ninety-four patients with unresectable metastatic colorectal cancer were selected from Sun Yat-sen University Cancer Center Database in the period between January 2007 and December 2013. All information was carefully reviewed and collected, including the treatment, age, sex, carcinoembryonic antigen, site of tumor, histology, cancer antigen 199, number of liver metastases, and largest diameter of liver metastasis. The univariate and multivariate analyses were used to detect the relationship between primary tumor resection and overall survival of unresectable stage IV colorectal cancer patients.

**Results:**

One hundred twenty-five received palliative resection, and 69 received only chemotherapy. Multivariate analysis indicated that primary tumor site was one of the independent factors (RR 0.569, *P =* 0.007) that influenced overall survival. For left-side colon cancer patients, primary tumor resection prolonged the median overall survival time for 8 months (palliative resection vs. no palliative resection: 22 vs. 14 months, *P =* 0.009); however, for right-side colon cancer patients, palliative resection showed no benefit (12 vs. 10 months, *P* = 0.910).

**Conclusions:**

This study showed that left-side colon cancer patients might benefit from the primary tumor resection in terms of overall survival. This result should be further explored in a prospective study.

## Background

In China, the fifth most commonly diagnosed cancer is colorectal cancer (CRC), which is also the fifth leading cause of cancer death for both male and female [1]. Almost 22% of all CRC patients already have liver or other distance organ metastasis at the first time of diagnosis [2]; however, radical surgery resection, which includes both primary and metastatic tumors, can only be performed in few of these patients. Depending on the data of National Comprehensive Cancer Network (NCCN), active chemotherapy should be recommended to unresectable stage IV CRC patients, and palliative surgical resection is considered for those who develop symptomatic disease, such as bowel obstruction, severe bleeding, or perforation [3]. The alternative strategy is to perform a palliative surgical resection of primary tumor first, which will prevent related complications; then, systemic chemotherapy should be administered to treat any metastatic disease. In the past, primary tumor resection was considered effective for relieving symptoms of primary tumor and improving the quality of life (QOL) for most metastasis CRC (mCRC) patients [4]. However, when combined with chemotherapy, recent studies have indicated that only a small percentage of patients suffered from primary tumor-related symptoms [5], including bowel obstruction, tumor perforation, and significant bleeding [6]. Therefore, until recently, the treatment strategy for asymptomatic unresectable stage IV patients was still under debate.

Surgeons prefer palliative primary tumor resection because it prevents the occurrence of primary tumor-related emergency events during chemotherapy. Further, some researches have reported that unresectable stage IV patients may benefit from primary tumor resection in terms of prolonging lifetime. By analyzing Surveillance Epidemiology and End Results (SEER) program database, Tarantino et al. found significant relationship between palliative surgery resection of primary tumor and better survival in unresectable stage IV CRC patients (HR 0.40, 95% CI 0.39–0.42; *P <* 0.001) [7].

In contrast, some oncologists prefer first-line chemotherapy for unresectable mCRC patients. However, some doctors are worried about the high rate of emergency events during chemotherapy and about postoperative complications after surgery, especially in patients experiencing a deteriorated overall condition, weight loss, and malnutrition [8]. Recent retrospective studies [9, 10] have demonstrated that less than 10% of patients who received chemotherapy, as their first step of treatment, would suffer from primary tumor symptoms requiring emergency treatment. Another study, also based on the NCI/SEER database, demonstrated a contrary result that palliative resection did not prolong survival [11].

Therefore, whether palliative surgical resection of primary tumor can favor overall survival (OS) is controversial and needs further assessment. No randomized controlled trial has addressed this clinical question. The CAIRO4 [12], NCT01978249 [13], SYNCHRONOUS [14], and GRECCAR-8 [15] trials are currently being performed, and the results will be available in a few years. The primary endpoint of the present study was to assess whether the site of CRC is a predictor of palliative resection in asymptomatic CRC patients with unresectable metastasis.

## Methods

### Patients

**Fig. 1:**
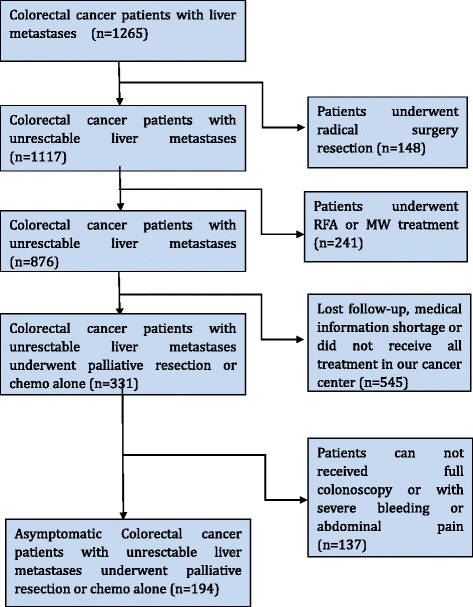
Flow chart for selecting asymptomatic colorectal cancer patients with unresectable metastasis who received palliative resection of the primary tumor or chemotherapy only

In this study, we assessed asymptomatic mCRC patients with liver metastasis at Sun Yat-sen University Cancer Center from January 1, 2007, to December 31, 2013. One thousand two hundred sixty-five CRC patients were selected and carefully reviewed, and 194 patients enrolled in the analysis according to the criteria of inclusion and exclusion (more details in Fig. 1). The inclusion criteria were 18–75 years old; pathologically confirmed colorectal cancer; unresectable synchronous metastases, as assessed by two experienced hepatobiliary surgeons; resectable primary tumor; performance status of 0, 1, or 2 depending on Eastern Cooperative Oncology Group (ECOG); no signs and symptoms of intestinal obstruction, perforation, or bleeding; and having received a full colonoscopy. The exclusion criteria were rectal tumors below 12 cm from the anal verge, primary tumor symptoms (severe bleeding, bowel obstruction, or tumor perforation), peritoneal or brain metastasis, or history of another primary cancer.

Written informed consents were provided by all patients before treatment. Colon cancer arising proximal to the splenic flexure was defined as right-side colon cancer (RSCC), and cancer arising distal to the splenic flexure was defined as left-side colorectal cancer (LSCRC). Clinicopathologic characteristics (patient age, sex, number of liver metastases, carcinoembryonic antigen (CEA), cancer antigen (CA199), diameter of the largest liver metastasis, active chemotherapy, and target agent) were compared between two groups. The protocol of this study was carefully reviewed and approved by ethics committee of Sun Yat-sen University Cancer Center.

### Statistical analysis

Continuous, normally distributed variables were compared between RSCC and LSCRC groups using independent *t*-tests, and dichotomous variables were compared between the groups using the Pearson chi-square test. The OS time was calculated from the confirmation of diagnosis to death or loss to follow-up (FU). The multivariate Cox proportional hazards regression model and the univariate Kaplan–Meier method were used to evaluate prognosis factors of OS. All statistical analyses were performed using SPSS software 19.0 (SPSS Inc. Chicago, IL, USA). A *P* value <0.05 was considered to be significant.

## Results

### Patient characteristics

One hundred ninety-four unresectable mCRC patients were selected and enrolled in this study. All 194 patients received a full colonoscopy and did not complain of primary tumor-related symptoms. Based on our grouping criteria, 50 patients were placed in the RSCC group, and 144 patients were placed in the LSCRC group. Between two groups, the clinicopathologic characteristics were well balanced. Detailed information for both cohorts of cases is listed in Tables 1 and 2.

**Table 1:** The clinicopathologic characteristics of all patients

Characteristic	RSCC	LSCRC	*P* value
Sex			0.107
Male	30 (60%)	104 (72.2)	
Female	20 (40%)	40 (28.8)	
Age	55.50 (23–75)	60 (20–75)	0.311
Liver metastasis			0.293
Single lesion	8 (16%)	15 (10.4)	
Multiple lesions	42 (84%)	129 (89.6)	
Diameter of liver metastasis			0.644
<3 cm	20 (40%)	63 (43.8%)	
≥3 cm	30 (60%)	81 (56.2%)	
CEA (ng/ml)			0.646
<200	40 (80%)	105 (72.9%)	
≥200	10 (20%)	39 (27.1%)	
CA199 (U/ml)			0.644
<35	20 (40%)	63 (43.8%)	
≥35	30 (60%)	81 (56.2%)	
Systemic chemotherapy			0.194
No	10 (20)	18 (5.6%)	
Yes	40 (80)	126 (94.4%)	
Target agent			0.970
None	39 (78%)	109 (75.7%)	
C225	5 (10%)	12 (8.3%)	
Avastin	4 (8%)	15 (10.4%)	
Both	1 (2%)	4 (2.8%)	
Other	1 (2%)	4 (2.8%)	

Data are presented as number (percentage) or mean (range)

*RSCC* right-side colon cancer, *LSCRC* left-side colorectal cancer, *CEA* carcinoembryonic antigen, *CA* cancer antigen

**Table 2:** The clinicopathologic characteristics of the patients who underwent palliative resection

Characteristics	RSCC	LSCRC	*P* value
Sex			0.179
Male	20 (58.8)	65 (71.4)	
Female	14 (41.2)	26 (28.6)	
Age	55.50 (23–75)	60 (20–75)	0.129
T stage			0.060
T1	0 (0)	1 (1.1)	
T2	3 (8.9)	7 (7.7)	
T3	6 (17.6)	29 (31.9)	
T4a	23 (67.6)	54 (59.3)	
T4b	2 (5.9)	0 (0)	
N stage			0.977
N0	12 (35.3)	33 (36.3)	
N1	10 (29.4)	25 (27.5)	
N2	12 (35.3)	33 (36.3)	
LVI			0.641
None	28 (82.4)	78 (85.7)	
Yes	6 (17.6)	13 (14.3)	
PNI			0.719
None	30 (88.2)	84 (92.3)	
Yes	4 (11.8)	7 (7.7)	
Histological types			0.270
Highly differentiated ADC	0 (0)	0 (0)	
Middle differentiated ADC	21 (61.8)	65 (71.4)	
Poorly differentiated ADC	12 (35.3)	20 (22)	
Undifferentiated ADC	1 (2.9)	6 (6.6)	
Regional LNs	13.15 (0–41)	9.74 (0–32)	0.012
Metastatic LNs	3.64 (0–21)	2.98 (0–19)	0.447
Liver metastasis			0.489
Single lesion	7 (20.6)	14 (15.4)	
Multiple lesions	27 (79.4)	77 (84.6)	
Diameter of liver metastasis			0.275
<3 cm	12 (35.3)	42 (46.2)	
≥3 cm	22 (64.7)	49 (53.8)	
CEA			0.646
<200	26 (76.5)	73 (80.2)	
≥200	8 (23.5)	18 (19.8)	
CA199			0.275
<35	14 (41.2)	44 (48.4)	
≥35	20 (58.8)	47 (51.6)	
Systemic chemotherapy			0.250
No	10 (29)	18 (19.8)	
Yes	24 (71)	73 (80.2)	
Target agent			0.836
None	28 (82.4)	72 (79.1)	
C225	3 (8.8)	5 (5.5)	
Avastin	2 (5.9)	6 (6.6)	
Both	1 (2.9)	4 (4.4)	
Other	0 (0.0)	4 (4.4)	

Data are presented as number (percentage) or median (range)

*RSCC* right-side colon cancer, *LSCRC* left-side colorectal cancer, *CEA* carcinoembryonic antigen, *CA* cancer antigen, *LVI* lymphovascular invasion, *PNI* perineural invasion, *ADA* adenocarcinoma, *LNs* lymph nodes, *LM* liver metastasis

### Treatment

Of the 194 patients, 91 patients in the LSCRC group and 34 patients in the RSCC group received palliative primary tumor resection (63.2 vs. 68%, *P* = 0.541), and 69 patients did not. Forty patients in the RSCC group and 126 patients in the LSCRC group received active chemotherapy (80 vs. 94.4%, *P* = 0.194). Most patients in both groups did not receive any target agent in this study (39 patients in the RSCC group and 109 patients in the LSCRC group, 78.0 vs. 75.7%, *P* = 0.970).

### Survival time

**Fig. 2:**
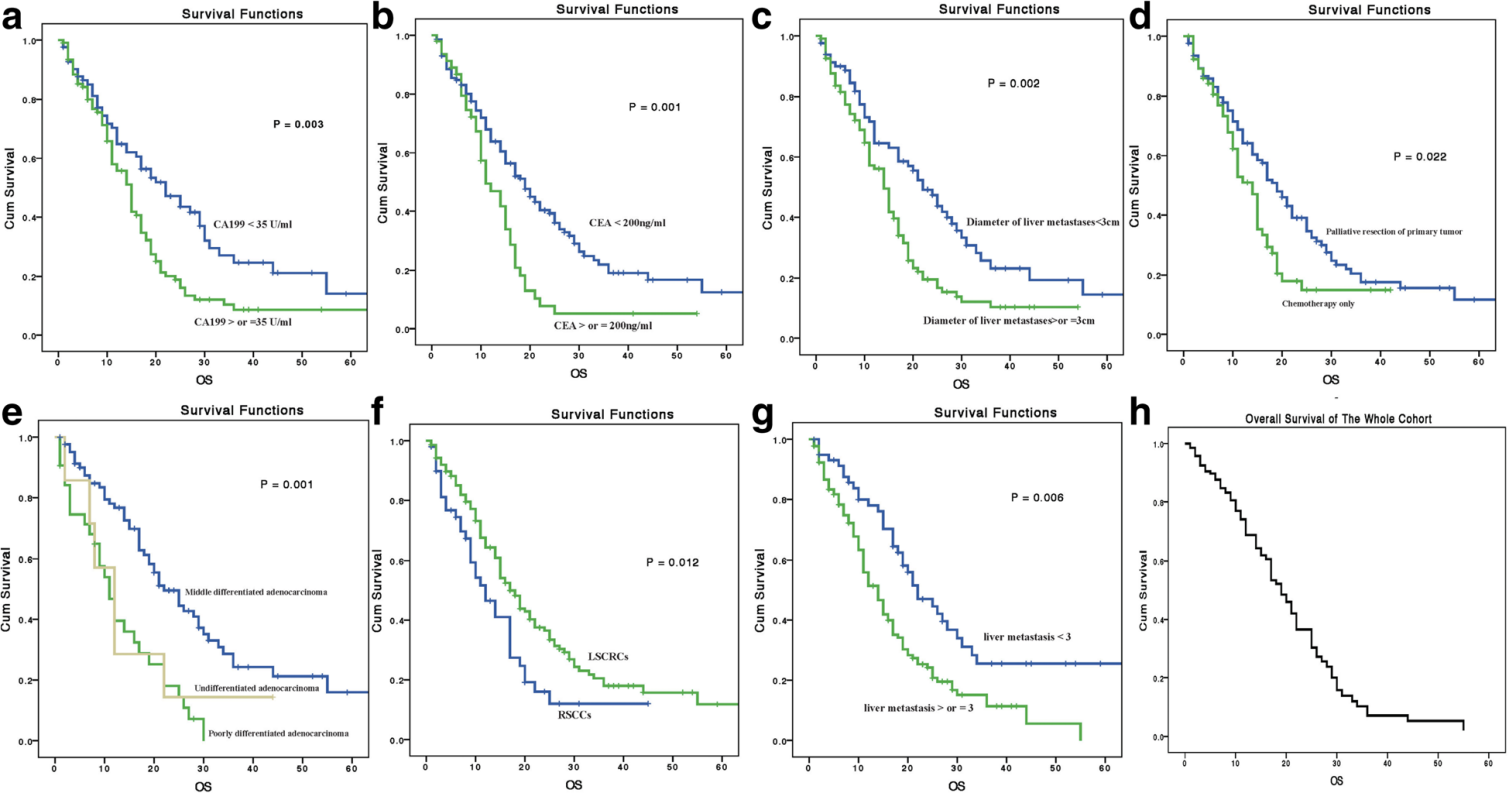
Kaplan–Meier curves for 5-year overall survival (OS) in different prognostic subgroups. **a** OS in different CA199 level groups. **b** OS in different CEA groups. **c** OS in different liver metastasis diameter groups. **d** OS in palliative resection and chemotherapy only groups. **e** OS in different histology groups. **f** OS in different primary tumor location groups. **g** OS in different number of liver metastasis groups. **h** Cox hazards regression analysis for 5-year OS in the entire cohort

**Fig. 3:**
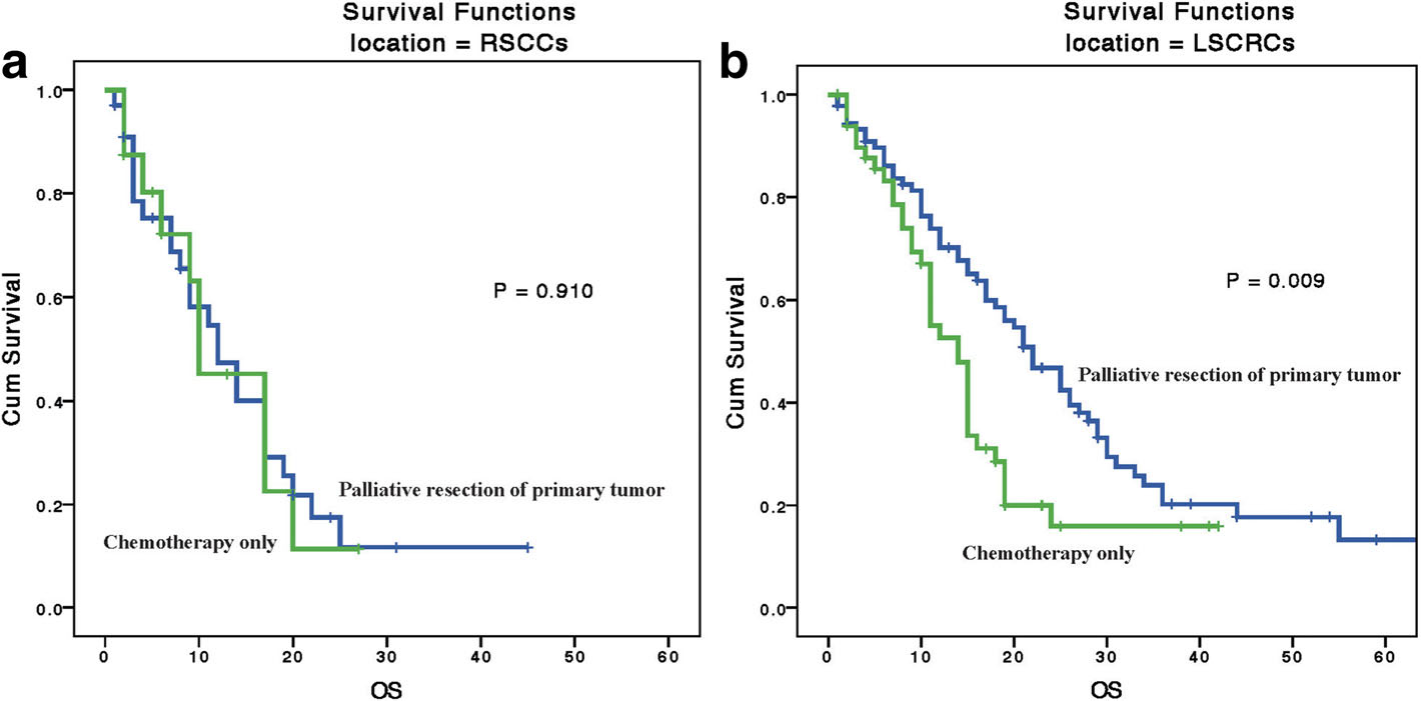
Kaplan–Meier curves for 5-year overall survival (OS) in different primary location subgroups. **a** Palliative resection shows no benefit in stage IV right-side colon cancer patients. **b** Stage IV left colorectal cancer patients show a benefit from palliative resection

The median duration of FU time of this study was 12 (1 to 79) months. In the RSCC group, the median survival times were 12 and 10 months for those with palliative resection and those without resection respectively, and in the LSCRC group, the corresponding survival times were 22 and 14 months respectively. The survival analysis of 5-year OS rate for the entire cohort was 11%, and the 3-year OS rate was 16% (Fig. 2). Univariate Kaplan–Meier analysis indicated that the location of primary tumor (RR 0.603, 95% CI 0.407–0.893; *P* = 0.012), diameter of liver metastasis (RR 1.766, 95% CI 1.233–2.530, *P* = 0.002), histological type (RR 1.733, 95% CI 1.273–2.357, *P* = 0.001), CEA (RR 1.926, 95% CI 1.309–2.834, *P* = 0.001), CA199 (RR 1.727, 95% CI 1.209–2.466, *P* = 0.003), number of liver metastases (RR 2.330, 95% CI 1.279–4.246, *P* = 0.006), and palliative primary tumor resection (RR 1.542, 95% CI 1.065–2.232, *P* = 0.022) were significant prognostic factors for OS in the entire cohort (Table 3). Based on a Cox hazards regression analysis, we found that the side of the primary tumor was an independent factor (RR 0.569, 95% CI 0.377–0.858, *P* = 0.007) that influenced OS. Other factors were the number of liver metastases (RR 2.134, 95% CI 1.420–3.205, *P =* 0.001), CEA level (RR 1.624, 95% CI 1.054–2.503, *P =* 0.028), and systemic chemotherapy (RR 0.582, 95% CI 0.350–0.968, *P =* 0.037). Palliative resection of the primary tumor showed no benefit in terms of OS (RR 1.034, 95% CI 0.973–1.098, *P =* 0.285) (Table 3; Fig. 2) in the entire cohort. In subgroup analysis, univariate analysis suggested that in the LSCRC group, primary tumor resection improved OS by 8 months (palliative resection vs. no palliative resection: 22 vs. 14 months, *P =* 0.009) (Fig. 3); however, in the RSCC group, palliative resection showed no benefit in terms of OS (12 vs. 10 months, *P =* 0.910) (Table 4, Fig. 3). Cox hazards regression analysis indicated that in the RSCC group, the only independent factor influencing OS was histological type (RR 0.313, 95% CI 0.134–0.729, *P =* 0.02). In the LSCRC group, the OS predictors were the histological type (RR 0.346, 95% CI 0.131–0.913, *P =* 0.032), metastatic lymph node number (RR 2.183, 95% CI 1.243–3.833, *P =* 0.007), and the primary tumor resection (RR 1.767, 95% CI 1.150–2.716, *P =* 0.011) (Table 4).

**Table 3:** Multivariate Cox proportional hazards regression analysis

	Overall survival			
	Univariate		Multivariate	
Variables	RR (95% CI)	*P* value	RR (95% CI)	*P* value
Primary location	0.603 (0.407–0.893)	*0.012*	0.569 (0.377–0.858)	*0.007*
T stage	1.384 (0.190–10.062)	0.748		
LVI	1.182 (0.819–1.708)	0.372		
PNI	1.456 (0.722–2.937)	0.293		
N stage	1.123 (0.267–4.717)	1.123		
Regional LNs	0.981 (0.948–1.016)	0.281		
Metastasis LNs	1.055 (1.006–1.106)	0.207		
Sex	1.260 (0.873–1.817)	0.217		
Diameter of LM	1.766 (1.233–2.530)	*0.002*	2.622 (1.618–4.248)	*0.001*
Neo-chemo	1.122 (0.654–1.925)	0.676		
Histological type	1.733 (1.273–2.357)	*0.001*	0.194 (0.075–0.501)	*0.001*
CA199	1.727 (1.209–2.466)	*0.003*		
Number of LM	2.330 (1.279–4.246)	*0.006*	2.134 (1.420–3.205)	*0.001*
CEA	1.926 (1.309–2.834)	*0.001*	1.624 (1.054–2.503)	*0.028*
Systemic chemo	0.738 (0.458–1.190)	0.213	0.582 (0.350–0.968)	*0.037*
Palliative resection	1.542 (1.065–2.232)	*0.022*	1.034 (0.973–1.098)	0.285

*RR* risk ratio, *CI* confidence interval, *LVI* lymphovascular invasion, *PNI* perineural invasion, *LNs* lymph nodes, *LM* liver metastasis, *Neo-chemo* neoadjuvant chemotherapy, *Chemo* chemotherapy, *CEA* carcinoembryonic antigen, *CA* cancer antigen

**Table 4:** Univariate and multivariate analyses for RSCCs and LSCRs

	Overall survival							
	RSCCs				LSCRs			
	Univariate		Multivariate		Univariate		Multivariate	
Variable	RR (95% CI)	*P*	RR (95% CI)	*P*	RR (95% CI)	*P*	RR (95% CI)	*P*
T stage	2.284 (0.205–25.41)	0.502			0.271 (0.064–1.141)	0.075		
LVI	1.219 (0.449–3.306)	0.697			1.198 (0.794–1.807)	0.389		
PNI	0.595 (0.211–2.441)	0.717			1.913 (0.806–.542)	0.142		
N stage	0.911 (0.267–4.717)	0.847			0.566 (0.131–2.445)	0.446		
Regional LNs	0.973 (0.913–1.036)	0.388			0.90 (.929–.013)	0.170		
Metastasis LNs	1.040 (0.960–1.126)	0.336			1.04 (0.987–1.112)	0.123		
Sex	1.363 (0.693–2.681)	0.369			1.143 (0.735–1.778)	0.553		
Diameter of LM	1.705 (0.833–3.491)	0.144			1.75 (.153–2.656)	*0.009*		
Neo-chemo	2.263 (0.931–5.504)	0.072			0.846 (0.424–1.686)	0.635		
Histological type	2.696 (1.327–5.476)	*0.006*	0.313 (0.134–0.729)	*0.02*	0.519 (0.203–0.923)	*0.001*	0.346 (0.131–0.913)	*0.032*
CA199	0.826 (0.426–1.621)	0.578			2.087 (1.371–3.177)	*0.001*		
Number of LM	1.864 (0.886–3.923)	0.101			2.022 (1.274–3.211)	*0.003*	2.183 (1.243–3.833)	*0.007*
CEA	1.075 (0.503–2.300)	0.852			2.310 (1.42–624)	*0.001*		
Systemic chemo	0.620 (0.267–1.442)	0.267			0.830 (0.462–1.492)	0.533		
Palliative resection	1.041 (0.500–2.179)	0.910			1.767 (11.15–2.716)	*0.009*	1.767 (1.150–2.716)	*0.011*

*RSCC* right-side colon cancer, *LSCRC* left-side colorectal cancer, *RR* risk ratio, *CI* confidence interval, *LVI* lymphovascular invasion, *PNI* perineural invasion, *LNs* lymph nodes, *LM* liver metastasis, *Neo-chemo* neoadjuvant chemotherapy, *CEA* carcinoembryonic antigen, *CA* cancer antigen

## Discussion

In the past, palliative resection was only considered for unresectable stage IV patients with complaints of the primary tumor-related symptom [16]. Recently, more surgeons have come to believe that palliative resection could prolong the survival of stage IV patients. Previous studies, most of which were retrospective and limited to single-institutional experiences [17–21], have indicated that primary tumor resection could benefit OS. However, most such research did not consider the effect that contemporary palliative chemotherapy regimens, such as FOLFIRI and FOLFOX, and the impact of biotherapy or molecular targeted agents, such as bevacizumab and cetuximab, have on the patient’s prognosis. Based on the NCI/SEER database, some studies could not repeat the promising results of the primary tumor resection of the palliative resection of primary tumor [11]. In this study, palliative resection did not show any OS benefit (RR 1.034, 95% CI 0.973–1.098, *P =* 0.285). Therefore, whether stage IV patients, particularly asymptomatic patients, can benefit from palliative surgical resection remains undetermined.

This research indicated that the side of primary tumor was one of the predictors of OS, which is consistent with the results of another study [22]. Multivariate analysis adjusted for known prognostic factors supported the side of primary tumor as an independent prognostic factor in mCRC, with LSCRC patients having better survival than RSCC patients (RR 0.569, 95% CI 0.377–0.858, *P =* 0.007). This phenomenon may occur for the following reasons. First, RSCCs and LSCRCs have differences not only in their embryologic development and blood supply but also in macroscopic pathology and clinicopathologic parameters [23, 24]. LSCRCs are easier to diagnose at an early stage because they are more likely to suffer from obstruction and other related symptoms because of typically infiltrating and constricting lesions that encircle the lumen, whereas most RSCCs are always present with mild and occult symptoms, which may delay their diagnosis [23]. Second, poor pathological features may be another reason for the difference between RSCC and LSCRC because poorly differentiated adenocarcinoma is more likely to be seen in RSCCs compared with LSCRCs [25]. Third, different molecular features may also be a factor. Some studies have reported that more Braf, Kras mutation, and MSI-H occurred in RSCCs [26, 27]. Finally, RSCCs are typically present with a more aggressive biological behavior compared with LSCRCs [28, 29]. These may all be possible reasons that affect the prognostic difference between RSCCs and LSCRCs. However, using such a differentiation has been shown to be unsuitable for patients who have undergone hepatic R0 resection [30].

Our study did not suggest that all patients in the cohort would benefit from palliative resection for primary tumors, and the result was coincidently similar to a Cochrane systematic review, where comparable outcomes were observed between the resected and unresected groups [31]. In a subgroup analysis, palliative resection can improve OS only for LSCRCs but not for RSCCs. A possible reason is that LSCRC patients are more easily to experience bowel obstruction, bleeding, and tumor perforation and to have worse outcomes, so they may benefit from primary tumor resection. Another possible reason may be that LSCRCs are more likely to have less aggressive biological behavior and are genetically more stable and have diploid DNA content, infrequent allelic deletions, stable karyotype, and normal regulation of c-myc [32]; thus, primary tumor resection could effectively reduce the tumor burden and prolong the life for LSCRC patients. A third possible reason may be that RSCCs were more likely to have Kras gene mutation than for LSCRCs [27], which was a potential predictor for the effect of palliative resection [32]. We believe that this may explain why RSCCs can benefit from palliative resection.

Rectal cancer patients, who were lower than 12 cm from the anal verge, were not included in this study because chemoradiotherapy may be recommended for these patients. Low rectal cancer patients may suffer more from primary tumor-related symptoms, including bleeding, anal pain, and irregular bowel movement, and they are more willing to require primary tumor resection. However, very low rectal cancer patients may refuse surgery because of the possibility of colostomy. Because the inclusion of low rectal cancer patients may have resulted in bias, they were not included in this study.

The selection bias associated with the retrospective nature of the study is the most important limitation of this study. We only assessed patients who received all their anti-cancer therapy in our institution to ensure that all complications and medical examinations were carefully evaluated and recorded in detail. To minimize the selection bias for asymptomatic patients, only patients who underwent full colposcopy and did not have any complaints of severe bleeding or severe abdominal pain were selected for this study. However, surgeons were more likely to select patients with complaints of primary tumor-related symptoms or those who were likely to have primary tumor symptoms to resect the primary tumor. Additionally, surgeons were more likely to perform surgical resection for “more favorable” patients who had a better ECOG status. Because of the retrospective nature of the study, the results still need to be further explored. The limited number of patients was another limitation of this study. We have initiated a randomized prospective clinical trial (NCT02149784) to further assess our results.

## Conclusions

This study indicated that left-side stage IV CRC patients had a better prognosis than do patients with right-side CRC. No statistically significant difference had been detected between palliative surgical resection of primary tumor and chemotherapy for asymptomatic mCRC patients; however, palliative resection can prolong OS in patients with left-side CRC. A further study is warranted to assess our results.

## Abbreviations

ADA: Adenocarcinoma
CA: Cancer antigen
CEA: Carcinoembryonic antigen
CRC: Colorectal cancer
ECOG: Eastern Cooperative Oncology Group
FU: Follow-up
LM: Liver metastasis
LNs: Lymph nodes
LSCRC: Left-side colorectal cancer
LVI: Lymphovascular invasion
mCRC: Metastatic colorectal cancer
NCI: National Cancer Institute
OS: Overall survival
PNI: Perineural invasion
RSCC: Right-side colon cancer
SEER: Surveillance, Epidemiology, and End Results Program
